# Aerosolised surfactant generated by a novel noninvasive apparatus reduced acute lung injury in rats

**DOI:** 10.1186/cc7737

**Published:** 2009-03-04

**Authors:** Yu Sun, Rui Yang, Ji-gen Zhong, Feng Fang, Jin-jin Jiang, Ming-yao Liu, Jian Lu

**Affiliations:** 1 Department of Pathophysiology, College of Basic Medical Sciences, Second Military Medical University, 800 Xiangyin Road, Shanghai, 200433, China; 2 Department of Pediatrics, Changhai Hospital, Second Military Medical University, 800 Xiangyin Road, Shanghai, 200433, China; 3 Latner Thoracic Surgery Research Laboratories, University Health Network, Toronto General Research Institute and Department of Surgery, Faculty of Medicine, University of Toronto, TMDT/MaRS East Tower – 13th Floor, Room 707, 101 College Street, Toronto, Ontario, Canada M5G 1L7

## Abstract

**Introduction:**

Exogenous surfactant has been explored as a potential therapy for acute lung injury (ALI) and acute respiratory distress syndrome (ARDS). In the present study, a nebuliser driven by oxygen lines found in the hospital was developed to deliver aerosolised porcine pulmonary surfactant (PPS). We hypothesised that aerosolised surfactant inhaled through spontaneous breathing may effectively reduce severe lung injury.

**Methods:**

Rats were intravenously injected with oleic acid (OA) to induce ALI and 30 minutes later they were divided into five groups: model (injury only), PPS aerosol (PPS-aer), saline aerosol (saline-aer), PPS instillation (PPS-inst), and saline instillation (Saline-Inst). Blood gases, lung histology, and protein and TNF-α concentrations in the bronchoalveolar lavage fluid (BALF) were examined.

**Results:**

The PPS aerosol particles were less than 2.0 μm in size as determined by a laser aerosol particle counter. Treatment of animals with a PPS aerosol significantly increased the phospholipid content in the BALF, improved lung function, reduced pulmonary oedema, decreased total protein and TNF-α concentrations in BALF, ameliorated lung injury and improved animal survival. These therapeutic effects are similar to those seen in the PPS-inst group.

**Conclusions:**

This new method of PPS aerosolisation combines the therapeutic effects of a surfactant with partial oxygen inhalation under spontaneous breathing. It is an effective, simple and safe method of administering an exogenous surfactant.

## Introduction

Exogenous surfactants have been routinely used to treat preterm infants with neonatal respiratory distress syndrome (NRDS), reduce alveolar atelectasis, improve oxygenation and stabilise the status of the lung fluid system [1]. Surfactant administration has also been attempted for the treatment of adults with acute lung injury (ALI) or acute respiratory distress syndrome (ARDS) [2,3]. Instillation of bolus exogenous surfactant into the airway through endotracheal intubation or bronchoscopy is the conventional way of administering surfactant; however, this method can be associated with complications, such as bradycardias, changes in blood pressure, drug reflux and the need to re-intubate [4-10].

Inhaling an aerosolised surfactant is another method of exogenous surfactant administration [11,12]. Ultrasonic or jet nebulisers have been used to generate aerosolised surfactant. Although these approaches have proven to be efficient and safe in animal models [13-15], the therapeutic effects of aerosolised surfactant in human clinical trials have not been convincing. Most ultrasonic or jet nebulisers require the patient to have mechanical ventilation to deliver aerosolised surfactants, however, the improper use of ventilators may cause or enhance lung injury [16-18]. Recently, an aerosolised surfactant delivered by continuous positive airway pressure (CPAP) has shown beneficial effects in the treatment of NRDS without the need for mechanical ventilation [19-22]. These interesting results suggest that aerosolised surfactants inhaled by spontaneous breathing may be an alternative method of surfactant-based therapies.

Our previous studies demonstrated that intra-tracheal instillation of porcine pulmonary surfactant (PPS) reduced ALI in a variety of animal models [23-25]. In the present study, we have developed a new noninvasive method to deliver aerosolised surfactant. We hypothesised that aerosolised surfactant inhaled by spontaneous breathing may effectively reduce severe lung injury. The effects of aerosolised PPS delivered by this new method were evaluated in a rat model with severe ALI induced by oleic acid (OA).

## Materials and methods

### Surfactant preparation

PPS was isolated from pig bronchoalveolar lavage fluid (BALF) using a protocol modified from the one used by Enhorning and colleagues [26]. Briefly, PPS was extracted from BALF using sequential centrifugation, chloroform-methanol extraction and acetone precipitation. PPS contains more than 90% phospholipids and about 1% hydrophobic protein, mainly surfactant protein B and C. It is approved for use in clinical trials of NRDS treatments by the State Food and Drug Administration of China.

### Acute lung injury model and experimental design

This study was approved by the Institutional Ethics Committee (protocol No. M2008-004/20080123), following the guidelines of the National Institutes of Health for the care and use of laboratory animals. Male Sprague-Dawley rats (weighing 200 to 250 g) had their food withheld for 24 hours but were allowed free access to water. All animals were anaesthetised with intraperitoneal pentobarbital sodium (30 mg/ml; 0.25 ml per animal; additional doses of 0.1 ml when necessary; Grinsted Products, Brabant, Denmark). One carotid artery was cannulated and flushed with heparinised saline (100 u/ml) to collect samples for blood gas analysis.

ALI was induced using 20% OA (Second Chemical Agent Factory, Yixing, China) diluted with 0.1% BSA. All animals received a 1.0 ml/kg diluted OA injection (using a syringe with a No. 4.5 pedo-scalp needle) through a lingual vein for one minute. Rats were divided into five groups: PPS aerosol (PPS-aer, n = 16), saline aerosol (saline-aer, n = 16), PPS instillation (PPS-inst, n = 10), saline instillation (saline-inst, n = 10) and model (injury only, n = 10) groups.

### Administration of surfactant

**Figure 1:**
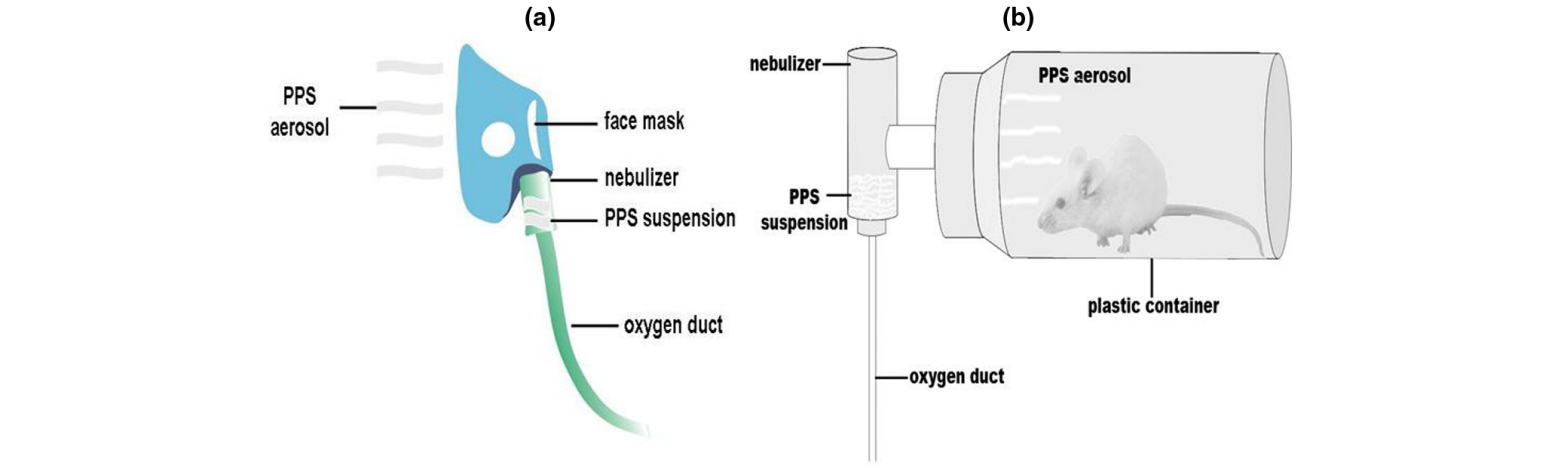
Experimental device for surfactant aerosol delivery.** (a)** A nebuliser is connected to an oxygen line and porcine pulmonary surfactant (PPS) in the nebuliser is converted to an aerosol by the jetting power of oxygen. **(b)** An anesthetised rat is placed in the plastic container and allowed to inhale the PPS aerosol by spontaneous breathing.

The nebuliser was driven by an oxygen line in the hospital ward and the flow rate used was 4.0 L/minute. When used clinically, the nebuliser can be connected to a facemask (Figure 1a); however, in the present experiment the anaesthetised rat was placed into a plastic container 30 minutes after the injection of OA. At the bottom of the container was a plastic plate, the anterior part of which was connected to the nozzle of a PARI LCD nebuliser (Pari Respiratory Equipment, Germany; Figure 1b). At the start of the experiment, the nebuliser was filled with a 20 mg/ml diluted PPS suspension given at a dose of 20 ml/kg. The first dose of PPS was nebulised over 20 minutes followed by 2 ml of saline nebulised over the next 10 minutes. This procedure was then repeated, giving a total amount of aerosolised PPS of 800 mg/kg. After the aerosol treatment, the animal was removed from the container and monitored until the end of the experiment. For the saline-aer group, an identical volume of saline was substituted for the PPS suspension. Animals were allowed to breath normally throughout the experiment except for the during the one hour of aerosolisation.

The rats in the PPS-inst and saline-inst groups were intubated after tracheotomy performed before OA injection. The rats in the PPS-inst group were instilled with 50 mg/ml PPS at a dose of 2 ml/kg via the endotracheal tube, and the saline-inst group was instilled with the same volume of saline. After instillation of PPS or saline, the lungs were filled with 2 ml of air four times.

### Assessment of surfactant treatment on acute lung injury

Arterial blood samples were taken before and every 30 minutes for four hours after the injection of OA. Arterial partial pressure of oxygen (PaO_2_), arterial partial pressure of carbon dioxide (PaCO_2_) and pH were measured with a DH-1830G blood gas analyser (Experimental Medical Apparatus Co., Nanjing, China). Respiratory rates were monitored at the time of blood sampling. At the end of the experiment, the survival rate of the rats was calculated. The animals were then sacrificed by exsanguination, and the lungs were removed and weighed. The lung index was calculated as the ratio of the wet lung weight to the body weight of the rat. Bronchoalveolar lavage was performed by lavaging 3 ml ice cold saline into the right lung. The lavage was repeated three times with about 90% of lavage fluid being recovered. The lavage fluid was immediately cooled to 4°C and centrifuged at 150 gfor 10 minutes. The total protein content and TNF-α concentration in the supernatant of BALF were measured with Lowry's protein assay and an ELISA kit (BioSource, Carlsbad, CA, USA), respectively. The left lung was expanded with air under 10 cmH_2_O pressure, fixed in 10% formalin and prepared for histological examination.

### Measurement of the particle size of aerosolised surfactant *in vitro*

A surfactant aerosol was diluted with air in a dilution bottle and the density of the surfactant aerosol was monitored with a laser aerosol particle counter (BCJ-1D; Institute of Optics and Fine Mechanics, Shanghai, China) and an oscilloscope (Model F123; Industrial Scopemeter, FLUKE Corporation, Everett, WA, USA). The size distribution of aerosolised surfactant in the dilution bottle was tested at three different flow rates (2.0, 4.0 and 8.0 L/minute).

### Surfactant phospholipid analysis in BALF

Lipids were extracted from the BALF of rats in the PPS-aer and saline-aer groups, and from a sham-operated normal control group using the method of Furue and colleagues [27]. The phospholipids were separated by thin-layer chromatography on pre-coated activated silica-gel type 60G plates. The plates were developed in a mixture of chloroform, methanol and water (at a ratio of 65:25:4, v/v). By exposure of the plates to I2 vapour, the lipids on the chromatograms were visualised and the spots of surfactant phospholipid on the plate were optically scanned.

### Histological examination

The lung sections were stained with H&E and examined with light microscopy. Lung injury was scored in a blinded fashion. Hyperaemia, atelectasis and neutrophil infiltration were scored as: 0 = minimal; 1 = mild; 2 = moderate; 3 = severe; 4 = maximal. Intra-alveolar oedema was scored as: 0 = absent; 1 = present.

### Statistical analysis

SPSS 12.0 statistical software (SPSS Inc, Chicago, IL, USA) was used. Data were analysed using the following tests: chi squared analysis for survival rates, Kruskal Wallis H test for histological data and one-way analysis of variance for multiple comparisons followed by Dunnett's test. P values of 0.05 or less were considered significant.

## Results

### Particle size of PPS aerosol

The size of the surfactant aerosol was measured three times at each flow rate (2.0, 4.0 and 8.0 L/minute) and the mean values and integrated count were obtained (Table 1). With a flow rate of 2.0 L/minute, 90.7% of the particles were smaller than 2.0 μm in diameter, although when the flow rates were more than 4.0 L/minute, almost all particles were smaller than 2.0 μm. The flow rate used clinically for oxygen delivery ranges from 3 to 5 L/minute, so most surfactant aerosol particles generated by this method should be 2.0 μm or smaller.

**Table 1:** Size distribution of porcine pulmonary surfactant aerosol particles

Particle diameter (μm)	Flow rate (L/minute)					
	
	2.0		4.0		8.0	
	
	MV	IC	MV	IC	MV	IC
≤ 2.0	90.7%	90.7%	99.8%	99.8%	100.0%	100.0%

2.0 to 5.0	2.3%	93.0%	0.2%	100.0%	0.0%	100.0%

≥ 5.0	7.0%	100.0%	0.0%	100.0%	0.0%	100.0%

IC = integrated count from three repeated measures; MV = mean value.

### Inhalation of PPS aerosol increased phospholipid content in BALF

**Figure 2:**
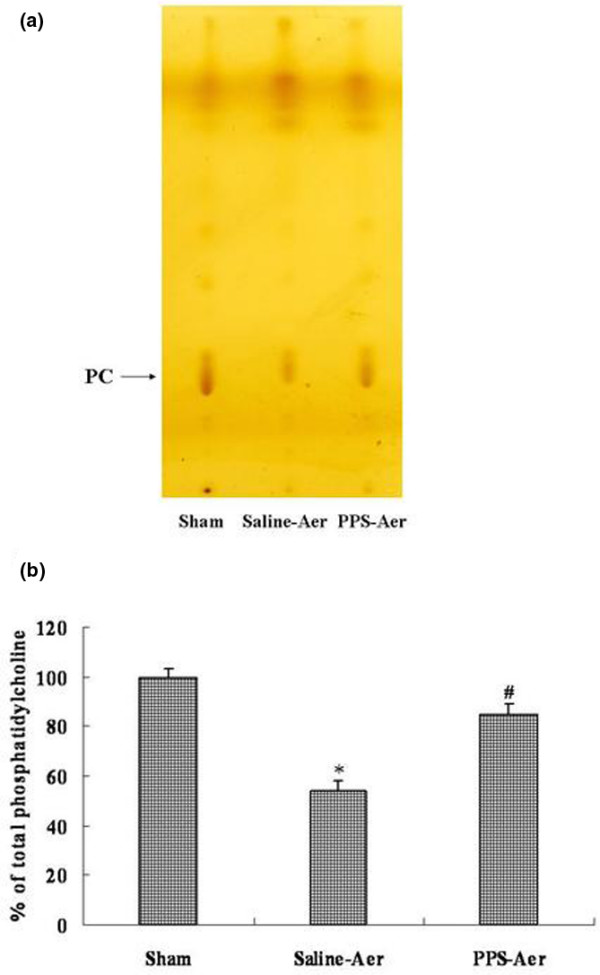
Inhalation of surfactant aerosol restored the phospholipid content in bronchoalveolar lavage fluid of rats with acute lung injury induced by oleic acid. **(a) **Phospholipids in the bronchoalveolar lavage fluid (BALF) separated by two-dimensional thin-layer chromatography. **(b)** Aerosolised surfactant restored phosphatidylcholine (PC) contents in BALF. Mean ± standard deviation from six animals per group. * *P* < 0.01 compared with the sham group. ^# ^*P *< 0.05 compared with the saline-aer group. PPS = porcine pulmonary surfactant.

We measured the phospholipid content in BALF using thin-layer chromatography. After injection of OA, phospholipids in the BALF of the saline-aer group significantly decreased, and the phosphatidylcholine content was only 54 ± 4.5% of that in the sham-operated group (*P* < 0.01; mean ± standard deviation). The phosphatidylcholine content in the BALF of the PPS-aer group was 85 ± 4.2% of that in the sham-operated group. This is significantly higher than that found in the saline-aer group (*P* < 0.05; mean ± standard deviation; Figures 2a, b). Inhalation of surfactant aerosol restored the loss of phospholipids in the lung.

### Aerosolised PPS improved the oxygenation function of the lung and decreased respiratory rates

**Figure 3:**
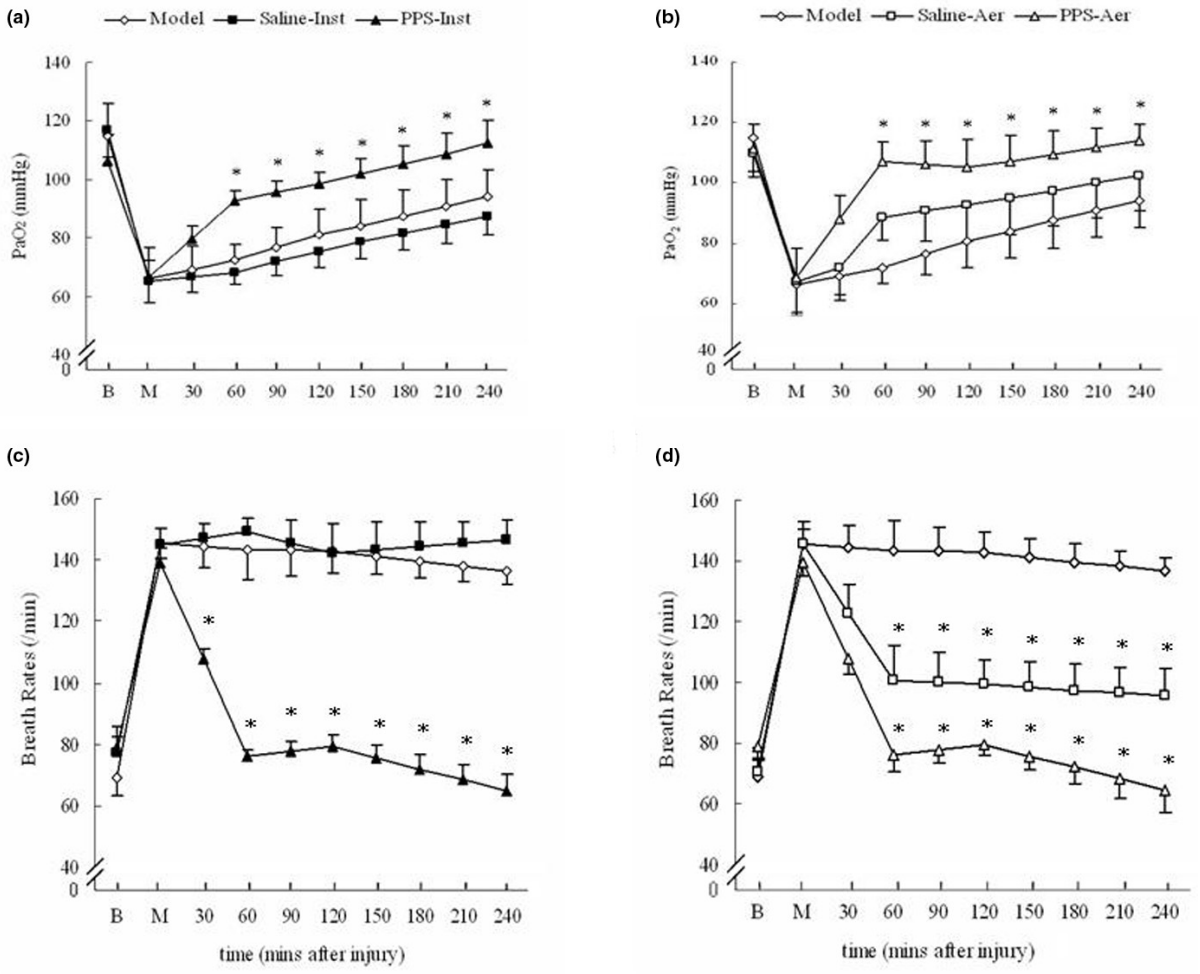
PPS administration improved recovery of PaO_2 _and decreased respiratory rates after acute lung injury was induced with oleic acid. **(a)** Arterial partial pressure of oxygen (PaO_2_) in instillation groups. **(b)** PaO_2 _in aerosolisation groups.** (c)** Respiratory rates in instillation groups. **(d)** Respiratory rates in aerosolisation groups. Mean ± standard deviation with different number of animals per group: model and saline-inst groups (n = 7), PPS-inst group (n = 10), saline-aer group (n = 13) and PPS-aer group (n = 16). * *P* < 0.05 compared with the model group. B = baseline; M = model, 30 minutes after oleic acid injection and the beginning of treatments; PPS = porcine pulmonary surfactant.

After OA injection, the PaO_2 _rapidly dropped from 110 to 64 mmHg (Figures 3a, b). Instillation of PPS (PPS-inst group) led to a gradual increase of PaO_2 _to about 110 mmHg after three hours (Figure 3a). The PaO_2 _in the PPS-aer group quickly increased to about 110 mmHg within one hour of PPS aesolization (Figure 3b). The PaO_2 _values in both the PPS-inst and PPS-aer groups were higher when compared with the model group after treatment (*P* < 0.05). The PaO_2 _values of the saline-aer group were also higher than those of the model group, although the differences were not statistically significant. The values of PaCO_2 _and pH among the groups showed no significant differences (data not shown).

Injection of OA accelerated respiratory rates from 70 to 80 breaths/minute to 140 to 150 breaths/minute in both the model and saline-inst groups. This acceleration was seen in these groups until the end of the experiment. In the PPS-inst group, tachypnoea induced by the OA injection returned to baseline (70 to 80 breaths/minute) within one hour of the treatment (Figure 3c). Inhalation of aerosolised saline decreased the respiratory rates in the saline-aer group to 100 breaths/minutes within one hour. The respiratory rates in the PPS-aer group were similar to the PPS-inst group, and returned to baseline (70 to 80 breaths/minute) within one hour (Figure 3d).

### Aerosolised PPS reduced lung oedema and inflammatory responses in the lung

**Figure 4:**
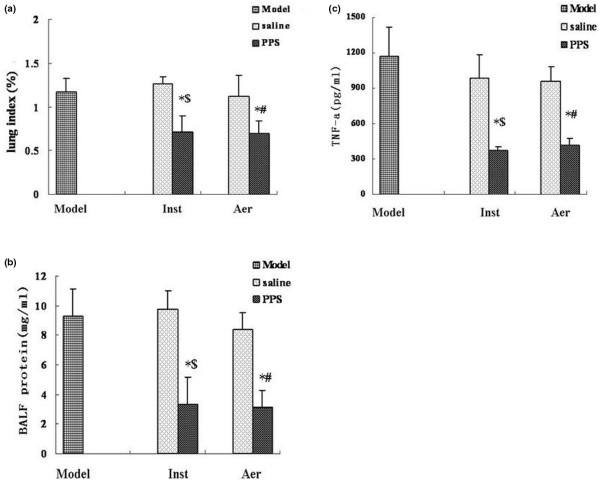
Exogenous surfactant reduced pulmonary oedema and inflammatory response. **(a) **Lung index (lung/body weight ratio), **(b)** total protein contents and **(c)** TNF-α concentration in bronchoalveolar lavage fluid (BALF). Mean ± standard deviation in animals in the model and saline groups (n = 7) and in the porcine pulmonary surfactant (PPS) groups (n = 10). * *P *< 0.01 compared with the model group. ^$ ^*P *< 0.01 compared with the saline-inst group.^# ^*P* < 0.01 compared with the saline-aer group. Aer = aerosolisation; Inst = intra-tracheal instillation.

As shown in Figure 4, a significantly decreased lung index (lung/body weight ratio; Figure 4a) and total protein content in BALF (Figure 4b) were observed in the PPS-aer and PPS-inst groups when compared with the model group and both groups that received saline (*P* < 0.01). ALI has been attributed to an excessive inflammatory response in the lung and treatment with exogenous surfactant significantly decreases the levels of pro-inflammatory cytokines [28]. TNF-α concentration in the BALF of the rats in the PPS-aer and PPS-inst groups was significantly reduced when compared with the model group and both groups that received saline (*P* < 0.01, Figure 4c).

**Figure 5:**
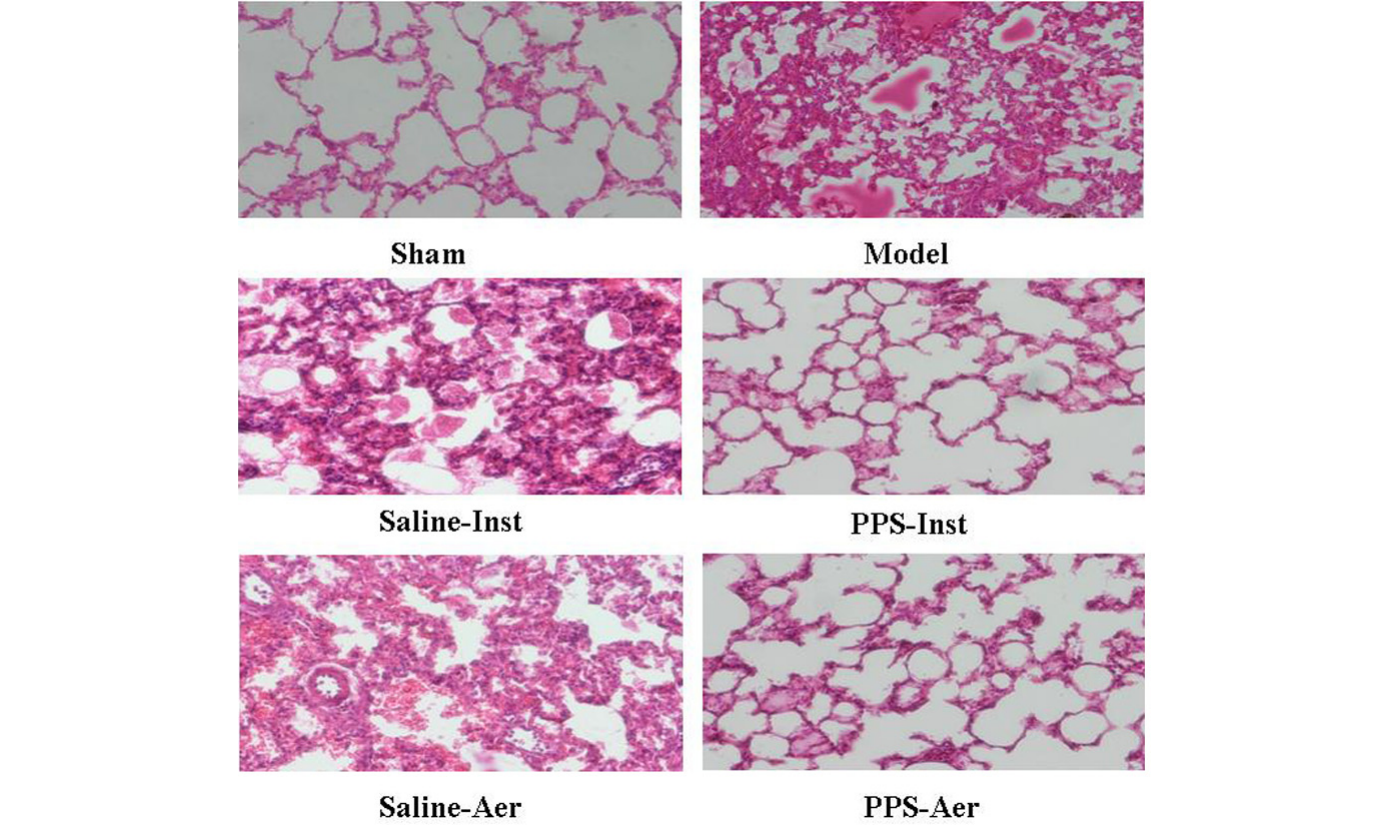
Exogenous surfactant administration reduced lung injury. Lung tissue slides were stained with H&E. Representative photomicrographs from each experimental group are shown (×200 magnification). PPS = porcine pulmonary surfactant.

The lungs in the model group and both groups that received saline appeared grossly swollen with diffuse haemorrhage in the lung tissue and pink exudates from the airway. Mean lung injury scores were significantly lower in the PPS-inst and PPS-aer groups than in the other groups (*P* < 0.05; Table 2). In particular, a reduction of intra-alveolar and interstitial oedema, and reduced haemorrhage and atelectasis were observed in the lung tissue of the PPS-treated groups (Figure 5).

**Table 2:** Exogenous surfactant administration in a rat model with oleic acid-induced acute lung injury

Group	n	Lung injury score
Model	7	9.5 ± 1.2
Saline-inst	7	10.8 ± 0.9
PPS-inst	10	4.6 ± 0.5*
Saline-aer	7	8.7 ± 0.8
PPS-aer	10	4.5 ± 0.4*

Data are mean ± standard deviation. * *P* < 0.01 compared with the model, saline-inst and saline-aer groups.

### Effects of PPS aerosol on survival

The survival rate in both the model and saline-inst groups was 70%. Three of the 16 rats in the saline-aer group died (81.25%) during the study, whereas the survival rate in both the PPS-aer and PPS-inst groups was 100% (*P* < 0.05, vs. the model and saline-inst groups; Table 3).

**Table 3:** Exogenous surfactant administration and survival rates in a rat model with oleic acid-induced acute lung injury

Group	Number of rats who survived (total rats)	Survival rate
Model	7 (10)	70%
Saline-inst	7 (10)	70%
PPS-inst	10 (10)	100%*
Saline-aer	13 (16)	81.25%
PPS-aer	16 (16)	100%*

* *P* < 0.05 compared with the model and saline-inst groups.

## Discussion

In the present study, we have developed a new noninvasive method of generating and delivering surfactant aerosol with a simplified nebulisation device driven by the oxygen line used in hospitals. We demonstrated the therapeutic effects of PPS aerosol on a rat model with OA-induced ALI.

The efficacy of aerosol delivery depends on the particle size. The average particle size of surfactant aerosols generated by ultrasonic or jet nebulisers is between about 4.5 and 3.5 μm, respectively [29,30]. In the present study, about 99.8% of the particles generated by our method at a flow rate of 4.0 L/minute were smaller than 2.0 μm, based on our calibration studies. It has been suggested that a particle size of 1 to 2 μm provides the best delivery of aerosols to lung peripheral regions. Particles between 2 and 6 μm in diameter are deposited in the central airways and those above 6 μm are depositied in the oropharynx [31]. A study by Minocchieri and colleagues in preterm infants showed that budesonide particles of 1.6 μm in diameter were optimal, with the lowest impaction and drug losses in the upper airways [32]. Although small particles might penetrate more deeply into the lung, they are also less affected by gravity and are more apt to be exhaled [31,33]. It is unknown how much of the inhaled surfactant particles were exhaled; however, we found a significantly increased phospholipid content in the BALF and a therapeutic effect on ALI in the PPS-aer group. These results indicated that at least some of the inhaled surfactant particles were deposited in the lungs. In future studies it will be necessary to estimate how much PPS is delivered into the lungs.

After injection of OA, respiratory rates increased from 70 to 80 breaths/minute to 140 to 150 breaths/minute and gradually returned to baseline within one hour in the PPS-aer group (Figure 3d). The average respiratory rate was about 110 to 120 breaths/minute during the one hour after aerosol administration. Assuming a tidal volume of 1.25 ml in rats weighing 200 to 250 g, the average passive minute volume should be about 140 to 150 ml/minute (1.25 ml × (110 to 120) breaths/minute = 140 to 150 ml/minute). As the flow rate of oxygen was 4.0 L/minute, about 3.5 to 3.8% of the total output of the nebuliser should have been inhaled by the rats. Other researchers have reported that about 4% of a radio-labelled surfactant aerosol, generated by nebulisers, reached the lung parenchyma with therapeutic effects on ALI [34-38]. Based on this estimation, about 25 mg/kg surfactant aerosol should have been deposited in the lungs. Obviously, this amount is much lower than the dose of surfactant given in the PPS-inst group (100 mg/kg). One plausible explanation is that the surfactant aerosol may have more success reaching the parenchyma of the lung, even in the presence of severe lung injury, than the bolus instillation of surfactant suspension.

It has been reported that administration of exogenous surfactant may not only prevent the lung from oedema by stabilising the fluid system in the lung, but may also inhibit the inflammatory response [28]. In this study, we showed a therapeutic effect of aerosolised PPS on OA-induced ALI, including reduced lung oedema, decreased total protein content and TNF-α concentration in BALF, ameliorated lung injury and increased survival rate. Aerosolisation of the surfactant by our new method was driven by oxygen, which may contribute to the quicker improvement of oxygenation and other therapeutic effects. Moreover, several studies have shown that saline aerosol inhalation provides certain beneficial effects on lung injury, probably due to the moistening of the airways [15,34]. Indeed, we noted the PaO_2 _and respiratory rates in the saline-aer group showed partial improvement when compared with the model group. It has also been found in previous studies that, in comparison with spontaneous breathing, the deposition of exogenous surfactant in lung tissue was impaired by mechanical ventilation in preterm newborn rabbits [17]. Using ^99m^Tc-labelled diethylenetriamine pentaacetic acid as a marker, MacIntyre and colleagues compared the deposition of surfactant in adult lung tissue. They showed that, although 11.9% of surfactant aerosol was deposited in the lungs of patients who were breathing spontaneously, only 2.9% of surfactant aerosol was deposited in the lungs of mechanically ventilated patients [39]. The new method developed in the present study relies on spontaneous breathing, so it may be more efficient in terms of delivering exogenous surfactant into the alveolar space. Therefore, the therapeutic effects observed in the PPS-aer group could be the combined effects of several beneficial factors. Further studies need to be performed with other ALI animal models to verify the therapeutic effects of aerosolised surfactant by this new method. Finally, a clinical trial of administration of surfactant by our method is under consideration.

## Conclusions

In summary, a new, noninvasive and effective method to generate and deliver aerosolised surfactant has been developed in the present study. It can be assembled in hospital settings containing an oxygen supply. It relies on spontaneous breathing without intubation and mechanical ventilation, and has been proven to be an efficient, simple and safe method of administering exogenous surfactant.

## Key messages

• Exogenous surfactant has been explored as a potential therapy for ALI and ARDS. In the present study, we developed a new noninvasive method to generate and deliver aerosolised surfactant with a simplified nebulisation device driven by a hospital oxygen line.

• In rats with ALI induced by OA, the therapeutic effects of aerosolised surfactant delivered by this new method were shown.

• Collectively, this new method is simple, and combines the therapeutic effects of surfactant with increased fraction of inspired oxygen. It relies on spontaneous breathing without intubation and mechanical ventilation, and is efficient and safe for the administration of exogenous surfactant.

## Abbreviations

ALI: acute lung injury; ARDS: acute respiratory distress syndrome; BALF: bronchoalveolar lavage fluid; BSA: bovine serum albumin; ELISA: enzyme-linked immunosorbent assay; H&E: haematoxylin & eosin; NRDS: neonatal respiratory distress syndrome; OA: oleic acid; PaO_2_: partial pressure of oxygen in arterial blood; PaCO_2_: partial pressure of carbon dioxide in arterial blood; PPS: porcine pulmonary surfactant; TNF: tumour necrosis factor.

## Competing interests

The authors declare that they have no competing interests.

## Authors' contributions

YS performed the animal experiments and wrote the manuscript. RY assisted in the animal experiments. JZ was responsible for the preparation of PPS. FF and JJ developed the new method for surfactant nebulisation. ML critically edited and revised the manuscript. JL was responsible for the design of the experiments, analysis of experiment results and the final revision of the manuscript. All authors have read and approved the manuscript.
